# A qualitative study utilizing Interpretative phenomenological analysis to explore disclosure in adolescents with turner syndrome

**DOI:** 10.1111/bjhp.12586

**Published:** 2022-02-14

**Authors:** Mhairi Nisbet, Rory O’Connor, Avril Mason, Elizabeth Hunter

**Affiliations:** ^1^ Mental Health & Wellbeing Academic Centre Gartnavel Royal Hospital University of Glasgow Glasgow UK

**Keywords:** turner syndrome, disclosure, diagnosis, endocrinology, dyadic interview, psychology

## Abstract

**Objectives:**

To explore the experiences of diagnostic disclosure and disclosure to others in adolescents with Turner syndrome (TS) and their parents/guardians. In addition, we sought to examine the impact of TS on girls with TS and their family’s lives.

**Design:**

A qualitative method utilizing interpretative phenomenological analysis (IPA) was employed.

**Methods:**

Five girls with TS and one parent/guardian of each girl completed dyadic and individual semi‐structured interviews. Interviews were audio recorded and analysed verbatim. Data were analysed in accordance with IPA guidelines, with a focus on the dynamic interactions within dyads.

**Results:**

Analyses identified three superordinate themes across the 10 participant accounts: communication and support, stigmatization of TS, and psychological consequences. Ten related subthemes are described alongside relevant quotations, highlighting a gradual process of diagnostic disclosure within families and wider health care systems. Both girls and their parents appeared to express a general desire to conceal TS from others, indicating possible TS‐related stigma. The results also demonstrate the varying impact TS can have within families.

**Conclusions:**

The findings provide insight into the lived experience of receiving a diagnosis of TS and the possible difficulties around disclosure to others. Potential recommendations for clinicians and parents include ensuring direct conversations about infertility occur within treatment and facilitating open, honest communication.

**Statement of contribution**

***What is already known on this subject?***

- Quantitative and qualitative research studies suggest individuals with Turner Syndrome have had either their full diagnosis, or some aspect of their diagnosis withheld from them.
- The current care guidelines for the lifelong management of Turner Syndrome recommends open and honest communication from health professionals, around the topic of disclosure.
- Prior research exploring adolescent’s experiences of discussing other health conditions or disorders such as HIV or Autism suggest teenagers are very reluctant to share their diagnosis with others, for fear of perceived stigma.

***What does the study add?***

- The study highlights the experience of disclosure in girls with Turner Syndrome from the unique, joint perspectives of both the girls and their parents/guardians. The innovative design adds to the preliminary research around adapting IPA across a range of diverse populations and settings, from multiple perspectives.
- In contrast to previous research, the majority of girls and their parents generally report a positive experience of diagnostic disclosure, describing a culture of openness and honesty within systems.
- Infertility is the most difficult aspect of Turner Syndrome to discuss and disclose, for both girls and their parents/guardians. Health professionals may also have stigmatised beliefs or attitudes around infertility, which could have an impact upon patient care.

## Introduction

Turner syndrome (TS) is a genetic condition which occurs in between 1:2000 and 1:2500 live born females (Apperley et al., 2018; Stochholm, Juul, Juel, Naeraa, & Gravholt, 2006). The condition is caused by the complete or partial absence of the second X chromosome. TS is characterized by short stature and infertility, alongside a broad range of other phenotypical characteristics such as an increased risk of renal and heart defects (Hall & Gilchrist, 1990). The age at which girls are diagnosed can therefore vary, from the antenatal period through to adolescence, depending on which features are evident (Apperley et al., 2018). A recent study which collected data from the medical records of TS patients between 1997 and 2016 found that the median age of diagnosis was 1.5 years, with a range of −0.5 (prenatal) to 18 years old (Swauger et al., 2021). However, if girls were not diagnosed within 1 year of life, the median age increased to 9 years, indicating diagnosis may be delayed if not identified early in life.

Preliminary research suggests that in the past, individuals with TS may not have always been informed of their diagnosis or may have had some aspects of the condition withheld from them. Gravholt et al. (2003) conducted a questionnaire study to explore the characteristics and risk factors for bone fractures in women with TS (aged 1–73 years old, median age 30). Unexpectedly, they discovered 45/322 participants they surveyed were unaware of their TS diagnosis. Further qualitative research by Sutton et al. (2005) explored the challenges experienced by females aged 7–59 with TS across the lifespan; many participants spontaneously reported that some aspect of the TS diagnosis had been kept secret from them. Secondary analysis examining the impact of secret keeping on girls and women with TS suggested parents are the most likely individuals to withhold diagnostic information from their daughters, particularly around the infertility component (Sutton et al., 2006).

Understandably, the age at which a girl receives her diagnosis shall impact upon how the condition is disclosed or discussed with her. Given that nearly half (46%) of girls receive their diagnosis within the first year of life, it is likely that their parents or guardians will have been involved in the disclosure process (Swauger et al., 2021). Those diagnosed later in life will likely have been informed by a health care professional, potentially at the same time as, or with their parents there for support. A joint account may therefore provide a more in‐depth understanding of the experience. As far as we are aware, there has been no prior research conducted which focuses on the joint perspectives of adolescent girls with TS and their parents. Lastly, TS is likely to impact upon individual members of a family as well as families as a whole and so joint interviews could provide important insight into similarities and/or differences in experiences. Advantages of dyadic interviews include the opportunity to bring interaction into the interview (Kitzinger, 1995) and expand the coverage of the research topic by participants sharing their point of view (Morgan, Eliot, Lowe, & Gorman, 2015). Conversely, previous research also indicates family members may not talk openly in front of one another to an interviewer if they worry about criticizing or raising sensitive topics which could hurt another’s feelings or damage the relationship (Corbin & Morse, 2003; Morris, 2001). Therefore, this study interviewed girls and their parents both together and individually.

Moreover, research exploring adolescents’ views and experiences of disclosing conditions such as autism and HIV highlighted teenagers are often reluctant to tell others about their diagnosis, for fear of being treated differently or experiencing stigma (Humphrey & Lewis, 2008; Michaud et al., 2009). As far as we are aware, no prior research has been conducted to explore how girls with TS feel about disclosing their condition to others. It has been recognized that when a child has a chronic illness, the whole family is affected (Stein et al., 2019). Therefore, clinicians are encouraged to adopt family‐centred models of paediatric care, which include the potential impact of the illness on parents/carers (Watts et al., 2014). Prior studies have identified considerable variety with regards to the level of impact a child’s chronic condition can have on family functioning and quality of life (Barlow & Ellard, 2006). To our knowledge, how TS impacts upon families as a system has similarly not yet been explored.

The primary aim of this study was to explore the nature of disclosure in adolescents with TS, including how girls learned they had TS, how they felt about disclosing their condition to others, and parents’ experience of discussing and potentially disclosing aspects of TS to their children. The secondary aim of this study was to explore how the diagnosis of TS impacted girls and their families.

## Methods

### Design

A qualitative approach was employed and interpretative phenomenological analysis (IPA) was the method of analysis. The aim of IPA is to explore in detail the processes through which participants make sense of their own experiences, focusing on their own perceptions, understanding, and views (Brocki & Wearden, 2006). This study aimed to develop a rich insight into parents’ and girls’ experiences of disclosure and the impact of TS on their lives. One of the key benefits of IPA is that it offers an in‐depth approach, detailing the processes of meaning making about a particular phenomenon. Categories to describe and explain the phenomena are derived inductively, through the process of initially analysing individual transcripts to examine the detailed experience of each person, to then search for common themes and points of similarity and divergence between cases. It was felt that IPA would therefore further add to the evidence base by gaining a richer understanding from the participants.

### Participants

Participants were five girls who had been diagnosed with TS and one of each of their parents/guardians. Participants were recruited from a paediatric and adult‐designated TS clinic covering a population in the West of Scotland. Participants who had received a diagnosis of TS, aged between 12 and 25 years old, with one parent/guardian who also wished to take part, were included in the study. Potential parents/guardians who wished to take part were included if they had a daughter aged between 12 and 25 years old diagnosed with TS. The study invited either one or both parents to take part; the parents who participated were those who were available to attend the interview, and tended to be the person who accompanied their daughter to her usual TS clinic appointments. Those who did not speak English or those with a significant learning disability (LD) were excluded (participants who do not or did not attend mainstream school were not included). Those with a significant learning disability were excluded because it is possible they might not have been aware of certain aspects of the condition, for example, the infertility component. Taking part in the interviews would not have been an appropriate setting for families to disclose or discuss potentially sensitive aspects of TS for the first time. Individuals with a significant learning disability may also experience difficulties with communication, which could impact their ability to understand the questions being asked during the interview. We excluded participants who do not speak English as they may also experience difficulties understanding the questions being asked, which could have impacted on the results of the study.

### Sample size

Due to the depth and detail of the analysis to understand a particular phenomenon, a small purposively selected population was deemed appropriate (Smith, Flowers, & Larkin, 2009). We therefore aimed to interview between six and eight girls alongside six and eight parents/guardians. Similar qualitative studies interviewing dyads were recruited around the same number of participants (Akeson, Worth, & Sheikh, 2007; Maxted, Simpson, & Weatherhead, 2014).

### Ethical approval

Ethical approval was obtained from the West of Scotland Research Ethics Service (ref: 19/WS/0132), and with permission from participants, all interviews were recorded using a portable audio‐recording device and then uploaded to a secure server. The audio files were deleted from the device and each participant was assigned a unique code and pseudonym. Written informed consent was obtained from all participants, including parents/guardians. Written consent was provided by the parent/guardian of participants under the age of 16 years old, and assent forms were completed by the girls under 16 years old.

### Recruitment and procedure

The lead clinicians from the TS clinics invited all females under their care, who met the inclusion/exclusion criteria to take part (23 young people in total), alongside their parents via post. Participant information sheets were posted alongside the invitations. Participants could indicate consent by contacting a member of the research team prior to their appointment if they wished to take part. Alternatively, the lead clinicians asked all potential participants if they would like to take part when they attended the clinic. If they indicated they would like to take part, the principal investigator (PI) contacted them to provide any further information required and arrange a suitable time and date to attend the interview. All interviews were conducted within the Royal Hospital for Children, Glasgow.

One interviewer (PI) conducted semi‐structured, face‐to‐face in‐person interviews, lasting between 53 and 92 minutes, between November 2019 and January 2020. Girls and their parents/guardians were initially interviewed together using a dyadic interview approach. Both parties were then interviewed individually unless they chose to decline. The rationale for conducting interviews both jointly and individually was discussed prior to obtaining informed consent, and during the individual interviews, the PI asked each person whether there was anything else they would like to add or discuss. All interviews were transcribed verbatim. The interview schedule (Appendix S1) was developed by the PI, in consultation with the lead clinicians from the TS clinics and another researcher who is a clinical psychologist working within a Paediatric Clinical Psychology Service. To explore participants experience of diagnostic disclosure, the girls were asked ‘Can you tell me a bit about how you found out you had Turner Syndrome?’ and their parents/guardians were asked ‘Can you tell me how you found telling (participant) about the aspects of their condition?’. To investigate the experience of disclosure to others, girls were asked ‘How do you feel talking about your condition to others?’. Unfortunately, due to time constraints, we did not consult with the public or patients in the development of the interview schedule. We recognize this as a limitation to the study.

### Data analysis

Data were analysed using IPA in accordance with the steps and procedures outlined (Smith et al., 2009), before conducting an overarching analysis within each dyad, focusing on the interactions between participants (Morgan et al., 2015; Van Parys, Provoost, De Sutter, Pennings, & Buysse, 2017). To ensure concepts and themes were constructed from participants’ individual perspectives, the transcripts were initially analysed individually. Line‐by‐line coding was conducted to increase methodological rigour, the initial codes representing different levels of interpretation: descriptive, linguistic, and conceptual. Emergent themes were organized into three categories; ‘Individual with TS’, ‘Parent’, and ‘Both’ to reflect whether the interpretations were experienced by individual members of the dyad or both members. Each dyad’s emergent themes were finally integrated and analysed across all family units to produce overarching superordinate themes (Van Parys et al., 2017).

We acknowledge the dyadic approach utilized is not typical of IPA, in that researchers would usually seek out a single and reasonably homogenous sample of participants. However, a number of studies have used IPA to explore complex, systemic experiences from multiple perspectives (Burton, Shaw, & Gibson, 2015), and a recent article outlines a series of multiple perspective designs and analytic procedures using IPA, which can be adapted and used across diverse populations and settings (Larkin, Shaw, & Flowers, 2019).

### Reflexivity

Reflexivity has been established as one of the methods researchers use to ensure rigor, trustworthiness, and quality within qualitative research (Dodgson, 2019). The PI therefore maintained reflective notes throughout the data collection, transcription, and analysis, with the aim of continually refining the thematic process. The PI is a female, trainee clinical psychologist and prior to contacting potential participants, she had no relationship with them. Her background in clinical psychology training facilitated the interviews and data analysis, given her experience conducting assessments, building relationships with clients, and interpreting and analysing language within sessions.

Two of the five transcripts were separately coded by an independent investigator (trainee clinical psychologist) to reduce researcher bias and improve the quality of the study. We hope the inclusion of an additional investigator's viewpoint improves the credibility of the results through the process of triangulation; the codes were compared and discussed to reach an agreement, arguably increasing the scope and deepening the understanding of the interpretation (Tracy, 2010).

## Results

Five girls and five parents/guardians completed semi‐structured interviews with the PI. Each parent–child dyad was initially interviewed together and then separately, in accordance with the protocol. None of the participants declined the individual interview. Relevant participant characteristics are outlined in Table 1. Their names are pseudonyms.

**Table 1:** Characteristics of participants

Participant	Age (years)	Age at diagnosis (years)	Age at diagnostic disclosure (years)	Parent
Sarah	16	Birth	There was no one defining moment of diagnostic disclosure; Jack estimated Sarah was around 5 as this was when she began receiving growth hormone injections.	Jack
Mary	17	13	13	Jim
June	21	Birth	Jane estimated around 4 or 5.	Jane
Lisa	17	8	8	Amy
Erin	14	Birth	There was no one defining moment disclosure; neither Erin nor Sue could recall a specific age.	Sue

Three superordinate and nine related subthemes were identified; the results are presented in Table 2. Each superordinate theme and associated subtheme are outlined in detail, alongside relevant quotations to illustrate participants’ lived experience. Each subtheme indicates whether it represents the experiences of girls, parents, or both members of the dyad.

**Table 2:** Superordinate themes and related subthemes

Theme	Subtheme	Participant (Individual with TS/Parent/Both)^a^
Communication and support	Disclosure as a process	Both
	The process of acceptance	Both
	Open communication within support systems	Both
Stigmatization of TS	Avoid diagnostic disclosure	Both
	Infertility; the elephant in the room	Both
	I am not disabled	Individual with TS
	Separation between TS and the self	Both
Psychological consequences	Avoidant coping strategies	Both
	Anxiety and uncertainty	Both

^a^ Subthemes representing the experiences of individuals with TS, their parents, or both members of the dyad.

### Communication and support

#### Disclosure as a process

While the two girls diagnosed with TS aged 8 and 13 were informed by a health professional (HP), the parents of girls who were diagnosed at birth (3/5) reported being significantly involved in disclosing the condition to their daughter, and described disclosure as a gradual process over time:

It was done subtly and naturally all throughout, all throughout her life. (Sue)

Progressively disclosing TS appeared to be a conscious decision, aimed at minimizing potential distress, and increasing the likelihood of successful adaptation to the condition. Developmental age and stage were factored into the decision‐making process, in relation to when to disclose certain aspects of TS:

Some parts I suppose we told her at that age and some parts maybe a bit later. I mean with your children, you know infertility, you’re not going to tell an eight‐year‐old everything (Jack)

Four of five girls could not recall many specific memories around diagnostic disclosure and tended to seek the information from their parents, who, in turn, were able to provide much more detailed accounts. The lack of detail could signify that as children, the initial disclosure had minimal impact on their self‐concept, whereas for parents, recalling the disclosure appeared to be much more emotive topic, indicating greater significance. The girls appeared to develop an understanding and attach subsequent meaning to their condition gradually over time:

[infertility] I think when I was younger it never really mattered, it never really….came to me what it was, em, until I kind of started high school and I realised this is kind of quite a big thing. This could have a big impact on my life. (Lisa)

#### The process of acceptance

Both the girls and their parents similarly describe coming to terms with the diagnosis as a gradual process over time:

I’m slowly coming to terms with it (Sarah)

You need time to process it yourself (Mary)

The process of acceptance appeared to be endless, continually challenged by changes in the manifestation of TS, alongside shifts in what the condition means. After learning they had TS, initially, shorter stature appeared to be the primary concern for girls due to differences in height between themselves and their peers. The meaning attached to infertility seemed to gain greater significance as the girls progressed through adolescence, possibly due to feelings of loss and developing understanding and insight into potential future difficulties, should they wish to start a family. Living with TS could be viewed as a continually shifting process between living well with TS in the background versus TS in the foreground, dependent on age, developmental stage, and TS‐related health difficulties.

Families appeared to develop a range of approaches and philosophies as a system to facilitate the process of acceptance; these included specific strategies such as learning about the condition, alongside relational processes between family members. One parent spoke about the role that the acquisition of knowledge played in accepting her daughter’s diagnosis, which could stem from a reduction in uncertainty:

And then the more you learn about it the more you think, oh, oh right, oh I can deal with that. (Jane)

Lisa notes how the involvement of siblings in care can aid acceptance, possibly indicating a transition from perceiving illness as an individual experience to experiencing illness as a family:

I think another thing that’s really helped me in terms of like advice and the advice I’ve been given is having [sister] there at the appointments. And she knows so much about it…so like I know I can go to her as well. (Lisa)

#### Open communication within support systems

A systemic culture of openness within families was described by both girls and their parents, suggesting childhood illness may, at times, bring families closer together. Open communication within families appeared to reflect the transition to experiencing an illness as a family, within the wider process of acceptance:

I can talk about anything with them, with my turner’s syndrome (Sarah)

Both girls and their parents reflected on their experiences with HPs and four of five reported an overall positive experience of diagnostic disclosure and subsequent management of TS. Both members of the dyad cited openness and honesty as factors which facilitated a positive experience, signifying the importance of trust within the patient–clinician relationship:

[consultants] They’ve never sugar coated anything, em, they’ve always explained em eh like…if…like everything that was going on, like if my height wasn’t going so well, everything like that they’ve been really open and as my mum said from the get go, em, people have been open and honest (Lisa)

Openness and honesty within families and the wider health care system could reflect an intention to normalize TS‐related difficulties and the experience of illness in general. It could be the case that a systemic culture of open communication aids successful adaption to living with TS.

### Stigmatization of TS

#### Avoid diagnostic disclosure

Conversely, despite all participants reporting open communication within their immediate families and generally with HPs, both parents and girls described a desire to conceal their diagnosis from others. Girls appeared to be particularly avoidant in discussing their diagnosis with their peers, highlighting possible feelings of shame:

If the situation arises and they need to know, I’ll let them know. But otherwise I do kind of keep it to myself (June)

The main barrier to diagnostic disclosure for girls was a fear of being treated or perceived differently, suggesting the girls had formed outward identities which could, in some sense, be tarnished by the disclosure of TS. A quote from June has been used to best demonstrate below:

I’d be worried if once I’d let them know…they’d….they’d treat me in a different way, or something like that eh, [becomes tearful], I don’t want people’s perceptions of me to be….coloured by this (June)

Its possible girls are modelling their parents’ behaviours; most parents (3/5) subtly encouraged their daughter to hide their diagnosis from others and/or avoided disclosing to others out with the immediate family:

I mean, my dad, Erin’s granddad doesnae know. We, we didn’t, we haven’t, we’ve shared it with just us (Sue)

There appears to be discrepancies between dyads experiencing and valuing open, honest communication within the immediate family system, but feeling much more hesitant to discuss the condition out with the family. It could be the case that parents are attempting to protect their children from either real or perceived stigmatized beliefs of others, and these beliefs are unintentionally instilled into their daughters. The philosophies and beliefs within family systems are complex, and seem to simultaneously attempt to normalize TS and difference, while also reflecting underlying beliefs that difference or illness is something to hide or keep secret from others.

#### I am not disabled

The tendency to avoid discussing the condition with others could stem from experienced or perceived stigmatized beliefs around disability; all girls described a fear of being perceived as disabled by others due to TS. These fears could reflect stigmatized societal attitudes towards disability and illness. For Lisa and June, the belief that others may perceive them as disabled led them to overcompensate and strive to ‘prove’ to others they are able and equal:

I just kind of wanted to prove that I can….that I’m good at maths and I can do this and I can do that, all the things that I’m not supposed to be able to do. (Lisa)

June states she wants to be *‘treated fairly, a level playing field’*, which indicates she may have been underestimated by others due to her TS. The desire to ‘prove others wrong’ could represent a ‘fight’ against the diagnosis and related difficulties. However, for some girls, this could arguably be an attempt to destigmatize any automatic, negative illness‐related beliefs of others. The significant difference in how TS can manifest and present in individuals may be a meditating factor; if girls do not experience significant TS‐related health difficulties on a day‐to‐day basis, they may not perceive themselves as an ‘ill’ or ‘less able’ person. Mary discussed this in relation to the minimal impact she feels the diagnosis has had on her life overall:

I’ve lived with it for eh…my whole life and it never…impacted anything, I was never, you know, unable to do stuff. (Mary)

#### Separation between TS and the self

Both girls and their parents/guardians reported a strong feeling that TS did not define them as an individual, which could similarly be understood as a fight against the diagnosis, or alternatively reflect the minimal impact of TS on the girls’ identity:

Feel comfortable knowing that it’s just a part of you, it’s not kind of all of you, it’s just a small, pardon the pun [all laugh] but, eh, it’s just a wee bit of you, it’s not….all of you. (Lisa)

Sarah relates this to the universality of being a human being:

like I have turners syndrome but I’m just like….I’m just like them…in that I am a human (Sarah)

Families appeared to have developed shared attitudes and values towards illness, which centred around girls being ‘more than a syndrome’. During the dyadic interviews, this was interpreted as a way of providing reassurance and normalizing TS. These attitudes may reflect the desire to reject the view of themselves as an ill or disabled person, and instead focus on individual strengths and living life outside of the condition. Jane’s quote below best demonstrates this:

they’re not a turner syndrome, they’re a girl who happens to have turner syndrome (Jane)

In conjunction, both girls and their parents described a sense of separation between the self and TS, possibly to externalize the condition:

I don’t know when it stops being me and kind of more about the condition…so I think with that like, it’s trying to disassociate it, like people that I meet from being the condition. (Lisa)

This is not Sarah; this is the Turner Syndrome. (Jack)

Alongside interpreting certain behaviours or traits as directly related to TS, some parents also described having difficulty differentiating or separating what was TS and what was their daughter’s natural temperament:

We don’t, you know, is it turner syndrome? Is this how she would be naturally anyway other than being a bit tall, well nobody can answer that question because you wouldn’t know’ (Sue)

One parent reflected on the image that is portrayed of girls with TS, for example, on the Internet. She talks about the change in society’s perception of individuals with Down syndrome and states she feels there needs to be a similar shift in the way others perceive TS. This could be interpreted as stepping away from a ‘medical model’ of illness towards an individual difference model of acceptance within society.

#### Infertility; the elephant in the room

Infertility was highlighted as a particularly difficult aspect of TS to disclose or discuss by both members of the dyad, possibly due to infertility being perceived as a more intimate, personal topic, combined with perceived stigma:

[infertility] Everything else just kind of…leads into like an explanation or something that can be explained but then…I don’t really want to talk about that and then….because that feels more personal to me than the height or the hearing. (Lisa)

Infertility was often cited much later during the interviews, after growth or other TS‐specific difficulties, which could be indicative of subtle avoidance. Surprisingly, three dyads spontaneously reported HPs might also avoid discussing infertility with girls with TS and their families:

It was alluded to in the first appointment. (Jim)

Nobody’s actually ever sat down and had a conversation about it. (Amy)

Avoiding the topic of infertility further demonstrates the complex process of discussing a condition or illness. Alongside a fear of being perceived as disabled, stigmatized beliefs around infertility could also serve as a barrier to diagnostic disclosure to others.

### Psychological consequences

#### Avoidant coping strategies

A variety of individual coping strategies were discussed throughout the interviews, however, using avoidance to cope with TS was a common subtheme across both the girls and parents’ experiences. Four of the five dyads described feeling ‘lucky’; either because they had developed few TS‐related physical health difficulties or generally believing TS had not significantly impacted on everyday life:

We feel very very lucky it's affected her so little. (Jim)

Perceiving themselves as ‘lucky’ appeared to reflect an underlying hierarchy of illness, for example, I have ‘mild’ TS in comparison to others, which could be a strategy to further separate themselves from TS and avoid feeling like they belonged to a TS group. Four dyads tended to minimize any difficulties:

Lisa has been very fortunate with it, it was a tiny tiny mosaic on you know once of her chromosomes, it wasn’t as if it was like really bad. (Amy)

Sue describes deliberately concealing her daughter’s diagnosis from her teachers, to wait and see if they notice she has TS. Her teachers did not report any concerns which Sue appears to interpret as ‘if you can’t see it, it doesn’t exist’:

as I say, I put that to the test to the school, for to them to tell me, you tell me there’s something wrong, and that’s again why I didn’t tell my mum and dad at the time, and now just my dad obviously, em. You tell me, that’s what I was always waiting on, you tell me. And nobody’s ever told me. So. (Sue)

Jane similarly seemed to minimize June’s difficulties during the dyadic interview, however, when interviewed alone she wonders whether her daughter uses avoidance to protect her:

Aye she appears to be content but June will…June will tell you what she thinks you want to hear a wee bit. And she’ll say that everything’s fine, she’ll say that’s fine, but she’ll no want me to worry, she might be doing it to protect me. (Jane)

One of the main discrepancies between the dyad versus individual interviews was observed during the individual interviews with parents; parents expressed a greater level of concern about the potential impact of TS on their daughter’s lives. Minimizing TS‐related difficulties could be an attempt to protect their daughters from distress. It is possible both girls and their parents minimize difficulties to protect the other from distress, resulting in a cycle of avoidance.

#### Anxiety and uncertainty

Several girls (Mary, June, and Lisa) spoke about experiencing anxiety and engaging in unhelpful thinking patterns such as rumination, in relation to TS and the associated health difficulties and generally across day‐to‐day life:

Every time I went to one [appointment] before, especially endocrine, I just got myself into a big panic and that and…I just, it wasn’t a nice feeling. (Lisa)

Their anxiety was observed and confirmed by their parents. TS appeared to generate a great deal of uncertainty for families. The variety in clinical manifestations, the unpredictable nature of how the condition develops over time, and uncertainty for the future led to difficulties for both girls and their parents:

Again, it was the uncertainty of ‘there might be’ sort of thing, there might be a problem. (Jim)

Worries about their daughter’s futures became more apparent during the parent’s individual interviews, further emphasizing the desire to minimize difficulties or worries in front of their daughter, to shield them from distress:

What we do think about is obviously will it…affect her more, in later life? (Jim)

## Discussion

The current study investigated the experience of having TS, specifically focusing on the nature of disclosure and how the diagnosis impacts girls and their families. It explored these experiences from the perspective of both adolescent girls and one of their parents/guardians, and the analysis revealed three key themes.

### Communication and support

Consistent with previous qualitative research exploring disclosure in children with genetic conditions (Gallo, Angst, & Knafl, 2009), parents described disclosing TS to their children as a gradual process, dependent on developmental age and stage. These findings are consistent with cross‐sectional research indicating, in addition to emotional maturity, age acted as a key factor which influenced when parents would choose to discuss infertility with their daughters with TS (King, Plamondon, Counts, Laney, & Dixon, 2016).

Conducting joint interviews appeared to be particularly important in developing an accurate, comprehensive narrative around diagnostic disclosure and revealed differences in the emotional impact of receiving the initial diagnosis. The girls recalled few memories around diagnostic disclosure, frequently turning to their parents for detail, and in turn parents were able to expand upon the topic, often reporting significant distress and feelings of shock following diagnostic disclosure.

Gradual disclosure appeared to be used as a strategy to facilitate acceptance of the diagnosis, alongside the acquisition of knowledge and a shift towards experiencing illness as a family rather than an individual. Overall, three of the five dyads expressed the belief that TS did not currently significantly impact upon their day‐to‐day life. Due to the wide variability in clinical manifestations, it could be the case that these girls suffer from fewer TS‐related health difficulties and families have therefore adapted well to living with a health condition. These dyads may also be ‘living well with TS in the background’ within the process of acceptance due to their developmental age and stage. The two dyads which reported a greater impact of TS included two of the oldest girls (aged 22 and 17), which could be linked to a greater significance in the meaning attached to TS, for example, in relation to infertility. The culture of openness and honesty described within family and wider support systems may have also facilitated successful adjustment to TS; Robinson (2017) notes having an open support system and being able to ‘share the experience’ helps both individuals and their families successfully manage chronic illness.

In contrast to previous research (Sutton et al., 2006), none of the girls reported perceived secret keeping in relation to their diagnosis from either their parents or HPs; a culture of openness and closeness between parents and children was reported by all five dyads. Moreover, four of the five dyads reported a positive disclosure experience, citing honesty and openness as key contributing characteristics. These findings may reflect positive changes in the way clinicians and parents have approached the disclosure of TS over the last two decades. The results are consistent with recent guidance produced for the successful lifelong management of girls and women with TS, which emphasizes the importance of open and truthful communication from HPs (Turner & Hozjan, 2019).

### Stigmatization of TS

Conversely, both girls’ and their parents expressed a desire to conceal the TS diagnosis from others, which could be indicative of perceived or experienced stigma in relation to TS. It could be the case that perceived or anticipated stigma is more prevalent in girls with TS rather than actual experienced stigma; only one of the dyads reported experiencing discrimination or teasing as a result of having TS. In conjunction with qualitative research exploring the experiences of adolescents with visible and invisible chronic illness (Kaushansky et al., 2017), girls discussed the particular challenges around disclosure to peers. Kaushansky et al. (2017) identified perceived fear of rejection, pity, and fear of being perceived as vulnerable or different as key barriers to disclosure; these results are strikingly similar to barriers to disclosure cited in the current study, in line with the subtheme ‘I am not disabled’.

The tendency to avoid discussing TS with others could, in part, stem from a fear of being perceived as disabled; a distinct subtheme representing the experience of girls with TS demonstrated that they did not want to be perceived as having a disability, which could be linked to the perceived negative connotations around disability. Sociological research around disability stigma suggests historically, disability has been seen as a form of involuntary social deviance, which generates negative responses from others (Grue, 2016). The determination expressed by a few of the girls to ‘prove’ they are able could also be interpreted as a desire for normalcy; previous research has suggested young people diagnosed with a chronic illness can feel different from their peers and therefore strive to present themselves as ‘normal’ to protect or reinforce a non‐different identity (Benson, Lambert, Gallagher, Shahwan, & Austin, 2015).

The stigmatization of TS may coincide with both the girls’ and parents’ tendency to view the diagnosis as part of their self‐identity but in no way the whole of oneself. Perceptions of stigma due to illness can significantly affect individuals identity and sense of self (Kleinman, 1988). The girls and their parents could be, in some sense, compartmentalizing or externalizing the diagnosis, as a way of protecting the girl’s non‐different identity. The extent to which individuals felt TS was separate from their self‐concept seemed to vary slightly, reflecting differences in the perceived impact of the condition. Therefore, it could also be the case that girls who are not significantly impacted view TS as less meaningful and less integral to their sense of self.

Consistent with previous literature (Sutton et al., 2006), infertility was described as being particularly difficult to discuss/disclose. A subtle avoidance of infertility was also evident during the interviews. Surprisingly, three of the five dyads reported HPs showed signs of avoidance in relation to infertility. HP’s hesitation around discussing infertility could reflect underlying beliefs that infertility is shameful or taboo, thereby acting as a predisposing factor for families to form stigmatized beliefs. These findings are consistent with literature describing the social stigma (Ergin et al., 2018) and ‘secret stigma’ (Whiteford & Gonzalez, 1995) accompanying infertility. Stigmatized beliefs around infertility may also contribute towards the desire to hide or conceal the TS diagnosis from others.

### Psychological consequences

Although three of the five families reported little impact of TS on their lives, the majority of dyads minimized TS‐related difficulties, which was interpreted as an avoidant method of coping. Avoidance has similarly been highlighted as a common coping strategy used by adolescents with a range of health conditions such as diabetes (Iturralde, Weissberg‐Benchell, & Hood, 2017), asthma, and celiac disease (Oppenheimer, Krispin, Levy, Ozeri, & Apter, 2018). Avoidant coping may relate to the previously described normalcy theory; minimizing difficulties alongside concealment of the diagnosis could facilitate the compartmentalization of TS as separate from the self, in the pursuit to feel ‘normal’.

However, the PI recognizes that the 'Avoidant coping strategies' subtheme could reflect an underlying bias, stemming from her own life experiences and experience as a trainee clinical psychologist. She acknowledges she has an awareness of the potential impact of physical health difficulties on well‐being and therefore viewed participants as having existing underlying difficulties which were minimized during the interviews. These beliefs could reflect a 'medical model' of disability. An alternative interpretation could suggest that both girls and their parents genuinely do not perceive TS significantly impacts their daily lives, either due to having few TS‐related health difficulties or having formed adaptive, 'strength‐based' cultures within their family units.

Consistent with prior research around uncertainty due to illness (Brown & Graaf, 2013; Mishel, 1988), the wide spectrum in disease severity and unpredictable nature of how TS develops over time appeared to generate a great deal of uncertainty for families. Uncertainty about their daughter’s future and general concerns around TS became particularly apparent during parent’s individual interviews. The inclusion of both individual and joint interviews highlighted a circular pattern of protection between dyads; however, this was particularly evident in parents. It could be the case that parents felt if they expressed concerns about the future this would exacerbate their daughter’s anxiety or fear of being different from others, and therefore they especially minimize difficulties in their daughter’s presence as a method of protection.

### Limitations

We recognize this study included a wide age range of participants with TS. Although this study aimed to recruit a homogeneous sample, the age at which girls had been diagnosed with TS varied among participants, which may have impacted upon their accounts. Differences in the experience of disclosure were highlighted between girls diagnosed at birth and girls diagnosed in later childhood/adolescence, and this may also impact upon other areas of life.

Moreover, the families who volunteered to take part did so in the knowledge they would be asked to discuss the topic of disclosure together. The sample may therefore be biased towards families who have an existing culture of openness around the topic of disclosure. Individuals with poor or no relationships with their parents may also have been reluctant to take part. We excluded participants with a significant LD; it is possible that individuals with an LD and their parents may feel TS has had a much greater impact on their day‐to‐day lives. Lastly, excluding non‐English speakers is a further study limitation. Many studies suggest cultural differences can impact upon attitudes and behaviours towards the disclosure of paediatric illness; it is possible that non‐Western cultures in particular may have different experiences, attitudes, and beliefs around disclosure and TS, which have not been represented in this study (Rosenberg, Starks, Unguru, Feudtner, & Diekema, 2017).

### Implications

The results indicate HPs should ensure they initiate explicit discussions around infertility with girls and their parents. HPs may wish to reflect upon their own beliefs around fertility and their role in relieving girls of perceived stigma in relation to infertility; some researchers suggest clinicians have a duty to protect and counteract harm resulting from infertility stigma (Cook & Dickens, 2014). A question arises around whose responsibility it is to inform girls of the fertility difficulties associated with TS, be that clinicians or parents. It could be helpful for clinicians to raise this topic with parents, to agree upon how to navigate the disclosure of infertility and encourage parents to utilize a ‘drip feed’ approach. A similarly gradual process of discussing infertility with girls may facilitate successful adaptation.

Parents should be offered specific advice and support from the HPs involved in their daughter’s care, around how to gradually discuss the diagnosis of TS and infertility with their children. HPs could direct parents towards specific materials and resources to help facilitate these discussions, to normalize the many different ways of having a family (Durrett 2020). It may be useful to consider potential ‘higher risk’ developmental life stages, when TS‐related difficulties may become apparent, so as clinicians can provide additional support to families, for example, when girls start receiving sex education in school, during pubertal development, and if/when women with TS wish to start a family.

Understanding the positive impact of open, honest communication within family systems and wider support systems highlights the importance of establishing this approach from initial diagnosis, to build an effective foundation for continued care and management of TS. Girls and their parents might minimize TS‐related difficulties, which could arguably act as a barrier to help‐seeking behaviours; if families wish to portray a ‘normal’ identity, they may be less likely to engage with services or seek support. Consistent with current guidelines, this study recommends clinicians continually assess and monitor mood and psychosocial functioning and encourage girls and their parents to seek and utilize additional supports when necessary. Psychological interventions to support girls with TS to manage anxiety should perhaps be routinely offered, alongside counselling to their parents/guardians, to give parents the space to process and reflect upon how TS impacts them as a family. Clinical psychologists or family therapists may be well placed to provide these interventions. Parents and girls should be provided with as much information as possible to minimize uncertainty and anxiety. This study may also have wider implications around how society views TS and difference, suggesting that stepping away from a ‘medical model’ to a ‘strength‐based model’ or model of individual differences could benefit girls and their families.

When considering disclosure in future research, comparisons between girls diagnosed at birth and girls diagnosed in later childhood/adolescence may be helpful, as this likely impacts upon girls and their family’s experiences. Further research around how to enable disclosure of TS to others may help to inform future care guidelines; the inclusion of advice around how to disclose TS to others could thus become a standard element of TS management. Moreover, future research exploring clinicians' perceived barriers to discussing infertility may provide further insights into infertility stigma and provide recommendations around how clinicians can manage this.

## Conclusions

This research has provided an in‐depth account of the experience of diagnostic disclosure and the tendency to avoid disclosure to others, from the perspectives of girls with TS and their parents/guardians. The study has also highlighted variations in the level of impact of TS across families. Recommendations for clinical practice include explicitly discussing infertility with girls and their families and emphasizing the importance of open, honest communication. Future research to further explore how to enable girls to disclose their diagnosis to others may be of use.

## Conflict of interest

All authors declare no conflict of interest.

## Author contribution

**Mhairi Nisbet:** Data curation (equal); Formal analysis (equal); Investigation (equal); Methodology (equal); Writing – original draft (equal); Writing – review & editing (equal). **Avril Mason:** Conceptualization (equal); Data curation (equal); Supervision (equal); Writing – original draft (equal). **Elizabeth Hunter:** Conceptualization (equal); Supervision (equal); Writing – original draft (equal). **Rory O'Connor:** Methodology (equal); Resources (equal); Supervision (equal); Writing – original draft (equal).

## Supporting information


**Appendix S1**. Interview schedule.

## Data Availability

Data available on request due to privacy/ethical restrictions. The data that support the findings of this study are available on request from the corresponding author. The data are not publicly available due to privacy or ethical restrictions.
