# Supplementary material for: A qualitative study utilizing Interpretative phenomenological analysis to explore disclosure in adolescents with turner syndrome
Source: Br J Health Psychol. 2022 Feb 14;27(3):990–1010. doi: 10.1111/bjhp.12586 (PMC9545481; doi:10.1111/bjhp.12586)
Supplement: Supplementary file 1 — Appendix S1. Interview schedule. [file BJHP-27-990-s001.docx]

**Appendix 1. Interview schedule**

Demographic information shall be collected as part of informed consent.

**Opening**

1. (Establish Rapport) My name is X and I’m a ……..
2. B. (Purpose) I would like to ask you some questions about how you learned you had Turner Syndrome and what it’s like having the condition. I would also like to get some feedback from your parent/carer around the same topics.
3. C. (Motivation) I hope to use this information to get a better understanding of how girls are told about Turner Syndrome and the different aspects of the condition, to hopefully help other people in the future.
4. D. (Time Line) The interview should take between 45 - 60 minutes.

**Body**

1. **(Topic) diagnostic narrative**

*Opening Question:*

1. Can you tell me a bit about how you found out you had Turner Syndrome?

*Parent/carer Question prompts:*

- Can you tell me a bit about your experience of telling (participant) they had Turner Syndrome?
- How did you feel when telling (participant) about their condition?
- How do you feel when discussing TS with (participant)?
- Do you have anything to add around (participants) diagnosis?

*Prompts:*

- How old were you when you received your diagnosis?
- What do you remember being told about your diagnosis?
- How did you feel?
- What was good about it?
- What could have been better?
- Did you suspect that you were not given information about TS?

1. **(Topic) aspects of the condition**

*Opening question:*

1. Can you tell me what having TS means for you?

*Prompts:*

- Do you have any health problems related to TS?
- Do you know of any other health problems that others with TS might have?

1. **(Topic) what is disclosed to others e.g. peers**

*Opening question:*

1. How do you feel talking about your condition to others?
2. How do you feel talking about (participants) TS to others? (parent question)

*Prompts:*

- Who do you feel comfortable talking about it with?
- What aspects are easier to talk about?
- What aspects are harder to talk about?
- What would be your worries around talking about your condition to others?
- How did you feel when talking to X about TS?

1. **(Topic) Impact**

*Opening question:*

1. How has having TS impacted you and your life?
2. How has TS impacted you as a family?

*Prompts:*

- How does this impact you day to day?
- How does this impact your family day to day?

1. **Opportunity to speak without parent/guardian present and vice versa**

*Opening question:*

1. Would you like the chance to speak without your parent/guardian in the room?

If yes:

- Are there any topics you’d like to revisit or anything else you’d like to add?

If no:

**Closing**

Thank you very much for taking part.
